# PI-RADS v2.1: What has changed and how to report

**DOI:** 10.4102/sajr.v25i1.2062

**Published:** 2021-06-01

**Authors:** Robin Scott, Shalendra K. Misser, Dania Cioni, Emanuele Neri

**Affiliations:** 1 Department of Radiology, Lake, Smit and Partners Inc., Durban, South Africa; 2 Department of Radiology, Faculty of Health Sciences Medicine, College of Health Sciences, Nelson R. Mandela School of Medicine, University of KwaZulu-Natal, Durban, South Africa; 3 Department of Translational Research, Academic Radiology, University of Pisa, Pisa, Italy

**Keywords:** prostate carcinoma, PI-RADS, magnetic resonance imaging, technical parameters for mpMRI of prostate, assessment categories to stratify risk, structured reporting

## Abstract

Multiparametric magnetic resonance imaging (MRI) of the prostate has become a vital imaging tool in daily radiological practice for the stratification of the risk of prostate cancer. There has been a recent update to the Prostate Imaging-Reporting and Data System (PI-RADS). The updated changes in PI-RADS, which is version 2.1, have been described with information pertaining to the recommended imaging protocols, the techniques on how to perform prostate MRI and a simplified approach to interpreting and reporting MRI of the prostate. Explanatory tables, schematic diagrams and key representative images have been used to provide the reader with a useful approach to interpreting and then stratifying lesions in the four anatomical zones of the prostate gland. The intention of this article is to address challenges of interpretation and reporting of prostate lesions in daily practice.

## Background

Multiparametric magnetic resonance imaging (mpMRI) of the prostate has an important role in the diagnostic work-up and staging of patients with suspected prostate cancer.^1^ Prostate imaging and interpretation up until 2019 was based on Prostate Imaging-Reporting and Data System version 2 (PI-RADS v2) which provided clinical guidelines for mpMRI of the prostate. The aim of PI-RADS v2 was to create a global standard in the acquisition, interpretation, and reporting of prostate mpMRI examinations, as well as to enhance the detection, localisation and risk stratification in patients with treatment-naïve prostate glands.^2^ Numerous studies have ratified the value of PI-RADS v2 but have also shown inconsistencies and limitations especially relating to inter-observer variability for evaluation of the Transition Zone (TZ).^3^ In an attempt to reduce inter-observer subjectivity, the PI-RADS steering committee, recommended multiple modifications to PI-RADS v2 by employing a consensus based system. The system maintains the same framework of assigning scores to individual sequences, and using these scores, to calculate an overall assessment category for each patient. The updated version is termed PI-RADS v2.1 and provides an approach to interpretation and reporting.

## Introduction

Prostate cancer (PCa) is the second most common cancer in men worldwide. Two thirds of PCa cases are diagnosed in the more developed regions in the world.^2,4^ In South Africa the age-adjusted risk of being diagnosed with PCa is 30.96/100 000 and the lifetime risk in men is 1:2.^5^ Traditional diagnostic tests for detecting PCa, namely prostate-specific antigen (PSA) and transrectal ultrasound-guided (TRUS) biopsy, lack both sensitivity and specificity. Prostate-specific antigen is specific to the prostate gland but not to PCa. Transrectal ultrasound-guided biopsy can miss up to 30% of tumours and in approximately 30% of cases underestimates tumour aggressiveness.^2,6,7^

Imaging with MRI was initially used for loco-regional staging and computed tomography (CT) or bone scintigraphy for distant staging in patients with biopsy-proven cancer. Recent advantages in MR technology that allows both anatomical and functional imaging to be performed simultaneously, mpMRI, has improved our ability to both detect and characterise prostate tumours.^1^

In 2012 the European Society of Urogenital Radiology (ESUR) released a standardised prostate MRI assessment called PI-RADS. This was PI-RADS version 1 (v1). This established clinical guidelines for the acquisition, interpretation and reporting of mpMRI of the prostate in order to facilitate a greater level of standardisation and consistency.^1^ These guidelines were based on the calculation of points for the evaluation of each focal lesion with different sequences, namely T2-weighted imaging (T2WI), diffusion-weighted imaging (DWI), dynamic contrast enhanced (DCE) and selective spectroscopy.^1^

In 2015 these guidelines were updated (PI-RADS v2) by the American College of Radiology (ACR) and EAU Section of Urological Research (ESUR). In the updated version, spectroscopy assessment was not included and DCE imaging was rated less significant. Prostate Imaging-Reporting and Data System v2 introduced two important changes to PI-RADS v1: the concept of a dominant sequence for each prostate zone (DWI for the periphery and T2W imaging for the transitional gland) and the relegation of DCE to a tie-breaker role when a lesion remains indeterminate.^8^ It is important to note that PI-RADS only indicates the ‘probability’ of a clinically significant cancer with a 5-point evaluation for a focal lesion. Prostate Imaging-Reporting and Data System v2 led to a more cogent scoring system for assigning an overall score (1–5) for a lesion – with a score of 1 indicating a low chance of significant disease, and a score of 5 indicating a high likelihood of significant disease.

Although PI-RADS v2 has been taken up and used broadly worldwide, experience has highlighted ambiguities in the scoring and limitations in relation to inter-reader reproducibility. Prostate Imaging-Reporting and Data System v2.1 recommends several minor adjustments to try and simplify assessment and reduce inter-reader variability without changing the overall scope and principle of v2.^1^

## What PI-RADS v2.1 aims to do^3^

In patients with suspected PCa, PI-RADS v2.1 is configured to improve detection, accurately localise, characterise and risk stratify lesions in treatment-naïve prostate glands.

There are a number of aims of the PI-RADS v2.1 update. The basic acceptable technical parameters to perform prostate mpMRI have been defined. Simple and standardised terminology for concise reporting have been devised. This will assist radiologists in prostate MRI reporting and help reduce inter-observer interpretative variability. Assessment categories, summarising the level or suspicion of risk of a lesion have been calculated. The guidelines will stratify patients who would benefit from biopsy and management intervention versus an observation strategy of management. The use of data obtained from mpMRI can be used to guide and perform MRI biopsy of suspicious lesions. The streamlined and updated v2.1 will facilitate multi-disciplinary meetings and afford more effective communication between clinicians and radiologists.

## Major revisions in PI-RADS v2.1^3^

### Image data acquisition − Technical specifications:

- T2WI: It is recommended to perform a T2W sequence in the axial plane and at least one additional orthogonal plane.
- DWI: clarification of *b*-values to use for purposes of DWI acquisition and for apparent diffusion coefficient (ADC).
- DCE MRI: Temporal resolution < 15 s is advised to show early focal enhancement. 3D T1W gradient echo (GE) sequences are preferred.

### Clarifications in interpretation criteria:

- Further description of assessment of lesions in the central zone (CZ) and the anterior fibromuscular stroma (AFMS).
- Revision in criteria for T2WI scores of 1 and 2 in TZ.
- Revision in determination of overall assessment category in TZ.
- Revisions in criteria for DWI scores of 2 and 3.
- Clarification of the distinction between −ve and +ve enhancement on DCE.
- Clarification in measurement of the prostate volume.
- Revision to sector map.

### Biparametric magnetic resonance imaging (bpMRI)

This is prostate MRI without contrast enhancement. The PI-RADS committee suggests bpMRI be reserved for select clinical indications.^3^ If bpMRI is performed, and dynamic contrast enhancement data not obtained, the TZ evaluation remains unchanged. The PI-RADS assessment category for a finding in the peripheral zone (PZ) is primarily centred on the DWI score and the lesions that receive a score of 3 on DWI will not be upgraded. The proportion of men with PI-RADS 3 lesions will likely increase and PI-RADS 4 will decrease, hence changing the possibility of clinically significant PCa in these categories. This will require additional documentation and subsequently, pathway modifications for biopsy-naïve and prior-negative biopsy men.^3^ Multiparametric prostate magnetic resonance imaging is preferred over bpMRI in patients with hip implants where artefactual degradation of the images, especially DWI, may be anticipated.

## How to do it – with v2.1 modifiers

### Clinical considerations^3^

#### Timing of MRI after biopsy

An interval of 6 weeks or longer between biopsy and MRI (if feasible).

#### Patient preparation

At present, there is no consensus regarding any patient preparation issues.

Consider:

- Antispasmodic agents to reduce motion artefact from bowel peristalsis.
- Light enema preparation or laxative to empty distal sigmoid colon and rectum of faeces. The rectum should be evacuated prior to the MRI appointment.
- Advise patients to refrain from ejaculation for 3 days prior to the study to maintain maximum distension of seminal vesicles.

#### Patient clinical information^3^

- Recent PSA level, (preferably done within the preceding 6 weeks) and PSA history (if available).
- Date and result of biopsy. Number of cores, locations, Gleason scores of positive biopsies.
- Other relevant clinical history:
  ■ Digital rectal examination (DRE) findings
  ■ Medications (alpha-blockers, hormones or hormone ablations)
  ■ Prior prostate infection, pelvic surgery, radiotherapy
  ■ Family history

### Technical specifications^3^ (Table 1)

**TABLE 1:** Parameters for multiparametric prostate magnetic resonance imaging sequences utilised on a 3T scanner at the authors’ institution.

Sequence	FOV	Slices	TR	TE	Slice thickness
T1 axial	340	46	875	10	4 mm
STIR coronal	300	34	5560	82	4 mm
T2 sagittal	220	33	6000	101	3 mm
T2 coronal	200	26	7500	101	3 mm
T2 axial	200	26	7500	101	3 mm
DWI	200	20	4600	74	3.5 mm
Dynamic (T1 VIBE)	200	22	5.45	2.06	3 mm

DWI, diffusion weighted imaging; FOV, field of view; STIR, short-T1 inversion recovery; TE, echo time (ms); TR, repetition time (ms); VIBE, volumetric interpolated breath-hold examination.

#### Magnetic Field Strength. Can use both 1.5 T and 3T devices. The latter is preferred.

The PI-RADS v2.1 assessment requires good quality T2WI, DWI and ADC sequences as these are pivotal to the accurate grading of lesions. In addition, further sequences are required in the holistic evaluation of the patient with large field of view (FOV) T1WI and T2WI sequences for loco-regional spread, extra-prostatic invasion, osseous and nodal metastases. Magnetic resonance spectroscopy has no longer been supported by PI-RADS since v2 and as such, is no longer a required sequence. A 3-Tesla (3T) scan is recommended and has several advantages but these are balanced by some disadvantages that radiologists should be aware of.

#### Protocol detail

- Isotropic T2 images:
  ■ high resolution small field of view, in two planes
  ■ axial (straight axial to the patient or an oblique axial perpendicular to the long axis of the prostate) and
  ■ at least one orthogonal plane (either coronal or sagittal). We routinely also acquire the coronal plane
  ■ a slice thickness of 3 mm with no inter-slice gap
  ■ field of view up to 20 cm to include the margins of the gland and seminal vesicles.
- Diffusion-weighted imaging and ADC map calculation should be performed using:
  ■ a low *b*-value of 0 s/mm^2^ – 100 s/mm^2^ (preference for 50 mm^2^ – 100 mm^2^)
  ■ an intermediate *b*-value set at 800 s/mm^2^ – 1000 s/mm^2^
  ■ optionally, an additional *b*-value set in the range of 100 s/mm^2^ – 1000 s/mm^2^
  ■ a high *b*-value (≥ 1400 s/mm^2^) image set is also mandatory, preferably obtained from a separate acquisition or calculated using the above mentioned sequences (calculated from low and intermediate *b*-value images).
- T1 pre-contrast acquisition:
  ■ a large FOV T1W sequence, acquired in the axial plane, should be included to show the extended pelvis (lymph nodes, bones, etc.)
- Dynamic contrast enhanced, 3D T1W GE acquisition:
  ■ the axial plane is the preferred sequence
  ■ high spatial resolution is advantageous (demonstrates peri-prostatic veins, enables differentiation between the PZ and the TZ at the apex, better for evaluating the more homogeneous enhancement, typically seen in tumours)
  ■ temporal resolution of ≤ 15 s is advised.

## Prostate anatomy and revisions in the sector map

The prostate gland, from superior to inferior, comprises the base, the mid-gland and the apex.

It is divided into four histologic zones, namely: The PZ which contains 70% – 80% glandular tissue, the TZ which contains 5% the glandular tissue, the CZ which contains 20% of the glandular tissue and the AFMS which contains no glandular tissue.^3^

These zones are then further divided into 41 anatomical sectors for recording pathology:

- TZ-anterior and posterior (TZa, TZp on the left and right)
- PZ-anterior, postero-medial, postero-lateral (PZa, PZpm, PZpl on the left and the right)
- CZ at the base (CZ)
- AFMS
- Urethra

Findings must be recorded on the sector map. The sector map divides the prostate into 41 sectors:

- 38 in the prostate (19 on each side)
- 2 in the seminal vesicles
- 1 in the external urethral sphincter

**FIGURE 1:**
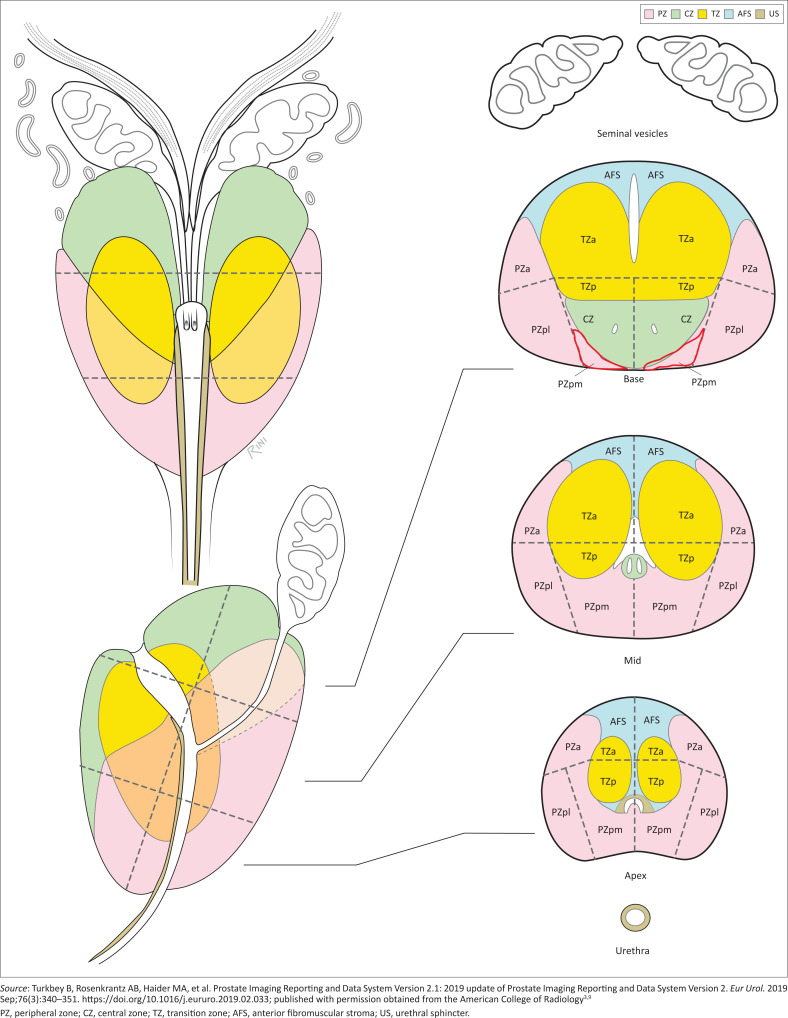
Sector zone map demonstrating the named 41 zones of the prostate.

Prostate Imaging-Reporting and Data System v2 had 39 sectors. Two additional sectors have now been added, namely the right and left posterior medial PZ (PZpm) at the base (see sector zone map in Figure 1).

## Image interpretation and how to report

### Image display

**FIGURE 2:**
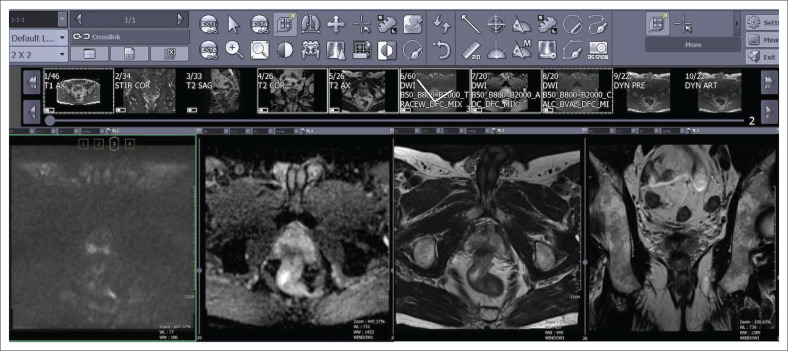
Workstation layout of four sequences used for multiparametric prostate magnetic resonance imaging evaluation of the prostate including (left to right) high *b*-value diffusion-weighted imaging, Apparent Diffusion Coefficient (ADC) map, axial and coronal T2-weighted imaging.

High resolution monitors (at least 3 megapixels) and a contrast ratio of at least 750:1 are required. Single or dual monitors may be used. How images are displayed is a personal preference and will depend on whether one or two monitors are in use. If two monitors are being used, divide each monitor into 4. Ideally, display images for ease of comparison between the important interpreting sequences, namely T2 images, High *b*-value DWI image, ADC map alongside one another. Layout of workstations is shown in Figure 2.

### Large FOV T1W sequence

Where available, this sequence should be used as an overview and to evaluate the soft tissues, the skeleton, lymph nodes and the prostate for signs of haemorrhage.

### Calculate the prostate volume

**FIGURE 3:**
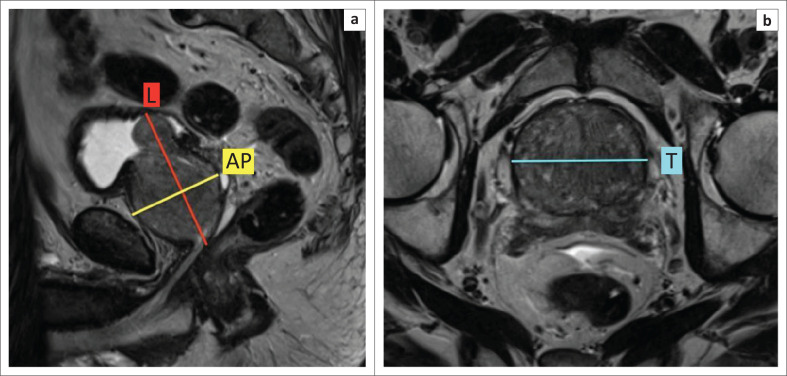
Recommended measurements for calculation of prostate volume at magnetic resonance imaging. Maximum longitudinal (L) diameter and maximum antero-posterior (AP) diameter measured on mid-sagittal T2-weighted imaging (T2WI) (a) and maximum transverse (T) diameter measured on axial T2WI (b).

This is calculated using the Ellipsoid Formula i.e. maximum longitudinal diameter {mid-sagittal T2} × maximum antero-posterior (AP) diameter {mid-sagittal T2} × maximum transverse diameter {axial T2} × 0.52.^3^ (Figure 3).

### Mapping lesions

Prostate cancer is often multifocal with the largest tumours yielding the highest Gleason Score and most likely contributing to extra-prostatic extension (EPE), and positive surgical margins. For PI-RADS v2.1, up to four lesions with a PI-RADS assessment score of 3, 4 or 5 may be assigned in each sector map. The ***index lesion*** should be identified. The index lesion is the one with the highest PI-RADS score. If there are two or more lesions with the same highest score, the index lesion will be the one with EPE. A smaller lesion with EPE (even if there are larger lesions) will be the index lesion. If there is no EPE then the largest lesion will be the index lesion. If there are more than four lesions, then only report the four with the highest likelihood of clinically significant cancer.

### Measurement of lesions

Multiparametric magnetic resonance imaging of the prostate underestimates tumour volume and extent when compared to histology. Report the largest dimension of a suspicious lesion on an axial image. If the largest dimension is on another plane, then report this measurement too. If the lesion on the axial image is not clearly delineated then measure the lesion on the best image (best plane). Peripheral zone lesions should be measured on ADC and TZ lesions on the T2W sequence. If the lesion is compromised on ADC or T2WI, then use the sequence that shows the lesion best.

### Peripheral zone interpretation (unchanged in v2.1)

**FIGURE 4:**
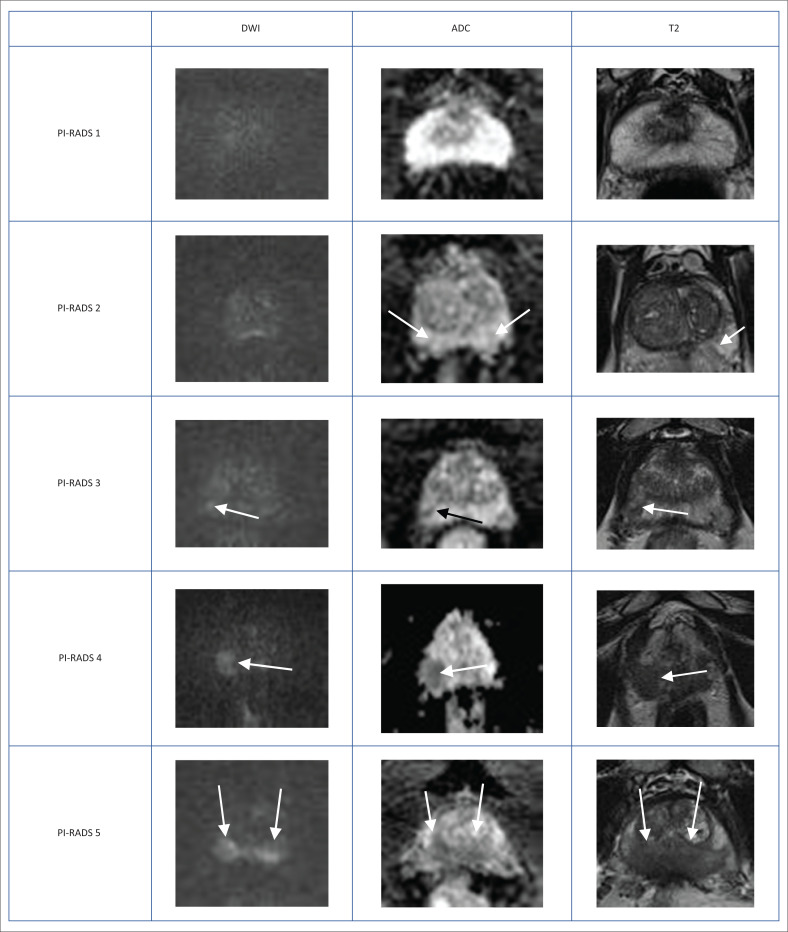
Typical peripheral zone Prostate Imaging-Reporting and Data System (PI-RADS) ^v2.1^ lesions. Peripheral zone (PZ) shows PI-RADS assessment categories with diffusion-weighted imaging (DWI) as the dominant sequence. (1) No abnormality (normal) on all sequences. (2) Iso-intense signal of the PZ on the high *b*-value DW image with an indistinct linear hypointense lesion on the Apparent Diffusion Coefficient (ADC) map (arrow) with corresponding T2W linear hypointense lesion. (3) Mildly hyperintense lesion of the PZ on the high *b*-value DW image with focal mildly hypointense lesion on the ADC map (arrow) and hyperintense non-circumscribed lesion on the T2W image. (4) Focal markedly hyperintense lesion (arrow) on the high *b*-value DW image with corresponding markedly hypointense signal intensity lesion on the ADC map. Lesion size < 1.5 cm in greatest dimension. T2W image demonstrates a circumscribed, homogeneous hyperintense mass confined to the prostate. (5) Same as (4) above but size > 1.5 cm in greatest dimension and could have extra-prostatic extension/invasive behaviour.

Although the main sequence is DWI, start by assessing the PZ on the T2W sequence; any T2 hypointensity needs further interrogation with DWI-ADC. The complementary sequence to DWI is DCE (Tables 2–4 and Figure 4).

**TABLE 2:** PI-RADS assessment of prostatic lesions according to diffusion-weighted imaging characterisation.

Score	Findings
1	No restricted diffusion. Normal on ADC.
2	Linear/wedge shaped restricted diffusion. Hypointense on ADC. Hyperintense on high *b*-value DWI.
3	Focal restricted diffusion. Subtle lesion. Discrete and different from background. May be markedly hypointense on ADC or markedly hyperintense on high *b*-value DWI, but NOT both.
4	Marked focal restriction of diffusion ≤ 1.5 cm in greatest dimension.
5	Marked focal restriction of diffusion ≥ 1.5 cm OR definite EPE / invasion of surrounding tissues.

*Source*: Turkbey B, Rosenkrantz AB, Haider MA, et al. Prostate Imaging Reporting and Data System Version 2.1: 2019 update of Prostate Imaging Reporting and Data System Version 2. *Eur Urol.* 2019 Sep;76(3):340–351. https://doi.org/10.1016/j.eururo.2019.02.033; American College of Radiology^3^

Note: Lesions with a score of three must be further evaluated on the DCE sequences to decide on the definitive PI-RADS score.

ADC, apparent diffusion coefficient; DWI, diffusion-weighted imaging; EPE, extra-prostatic extension; PI-RADS, Prostate Imaging-Reporting and Data System.

**TABLE 3:** PI-RADS features of prostatic lesions according to dynamic contrast enhanced characterisation in the peripheral zone.

Score	Findings
Negative	No early enhancement or diffuse multifocal enhancement NOT corresponding to the lesion of suspicion OR focal enhancement corresponding to a lesion with features of BPH on T2WI, including extruded BPH.
Positive	Focal and earlier than or contemporaneous enhancement of a suspicious lesion (corresponding to T2WI and/or DWI findings) relative to other normal prostatic tissue.

*Source*: Turkbey B, Rosenkrantz AB, Haider MA, et al. Prostate Imaging Reporting and Data System Version 2.1: 2019 update of Prostate Imaging Reporting and Data System Version 2. *Eur Urol*. 2019 Sep; 76(3):340–351. https://doi.org/10.1016/j.eururo.2019.02.033; American College of Radiology^3^

Note: If positive enhancement, then the lesion is PI-RADS 4. If negative enhancement, then the lesion remains PI-RADS 3.

BPH, benign prostatic hyperplasia; DWI, diffusion-weighted imaging; PI-RADS, Prostate Imaging-Reporting and Data System; T2WI, T2-weighted imaging.

**TABLE 4:** Combined evaluation of peripheral zone prostatic lesions using the diffusion-weighted imaging score, dynamic contrast enhanced score and T2 score to derive the overall PI-RADS grading.

DWI score dominant sequence	DCE score secondary sequence	T2 score	Overall PI-RADS
1	Any	Any	1
2	Any	Any	2
3	−ve	Any	3
3	+ve	Any	4
4	Any	Any	4
5	Any	Any	5

*Source*: Turkbey B, Rosenkrantz AB, Haider MA, et al. Prostate Imaging Reporting and Data System Version 2.1: 2019 update of Prostate Imaging Reporting and Data System Version 2. *Eur Urol.* 2019 Sep; 76(3):340–351. https://doi.org/10.1016/j.eururo.2019.02.033; American College of Radiology^3^

DWI, diffusion-weighted imaging; DCE, dynamic contrast enhanced; PI-RADS, Prostate Imaging-Reporting and Data System.

### Transitional zone interpretation (changed in v2.1)

**FIGURE 5:**
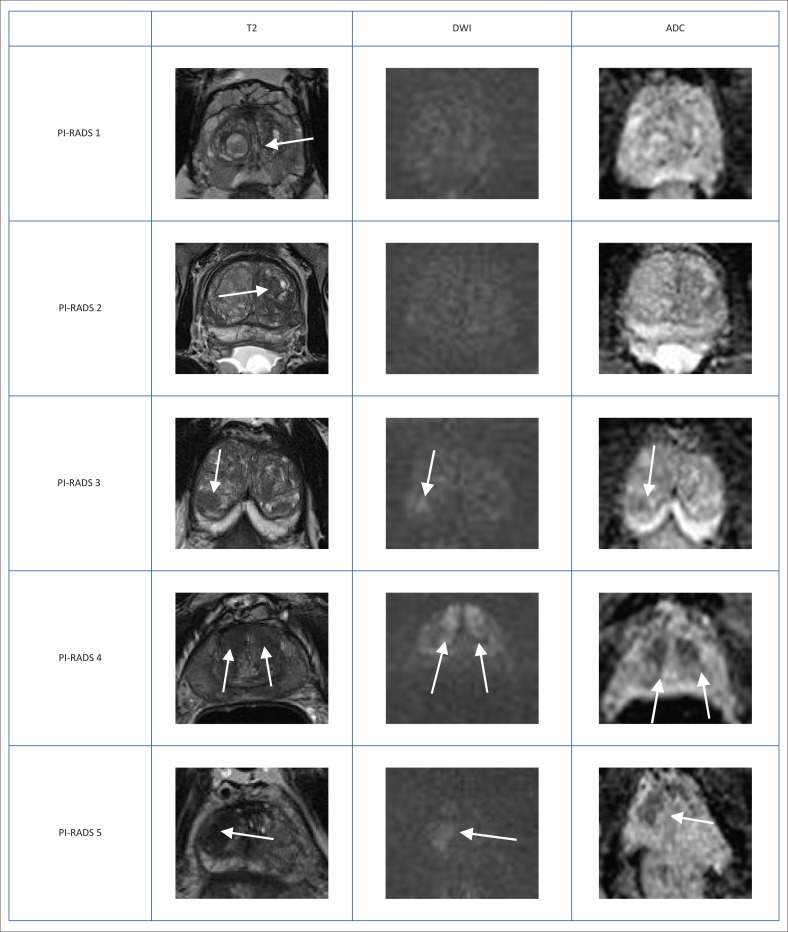
Typical Transition Zone Prostate Imaging-Reporting and Data System (PI-RADS) ^v2.1^ lesions. Transition Zone (TZ) shows PI-RADS assessment categories with T2-weighted imaging as dominant sequence. (1) Homogeneous intermediate signal intensity OR round, completely encapsulated nodule(s), that is, ‘typical nodule’. (2) Mostly encapsulated nodule (arrow) OR homogeneous circumscribed nodule without encapsulation OR homogeneous mildly hypointense area between nodules. (3) Heterogeneous, hypointense signal intensity with obscured margins. On Apparent Diffusion coefficient (ADC) map, focal (discrete and different from background) hypointense and/or focal hyperintensity on high *b*-value diffusion-weighted image. (4) Non-circumscribed (arrow) homogeneous moderately hypointense lesion on T2-weighted image. Must be < 1.5 cm in its greatest dimension. On ADC map, markedly hypointense and markedly hyperintense on high *b*-value DW image. (5) Same as (4) above but >1.5 cm in greatest dimension or extra-prostatic extension/invasive behaviour.

**FIGURE 6:**
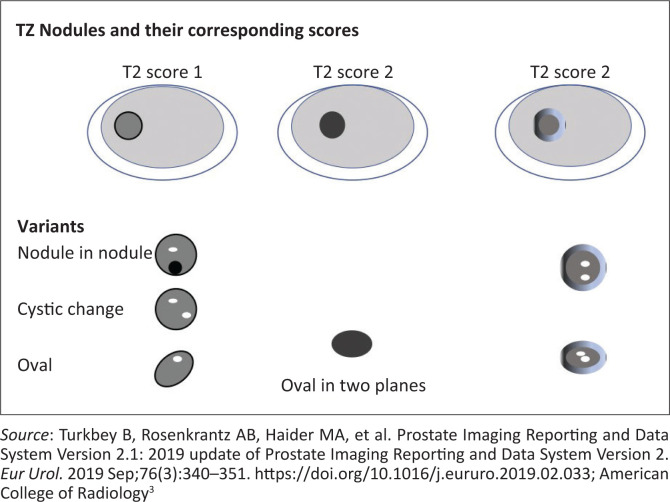
Schematic diagram of transition zone nodules, the corresponding T2-weighted imaging scores and description of acceptable variants.^10^

**FIGURE 7:**
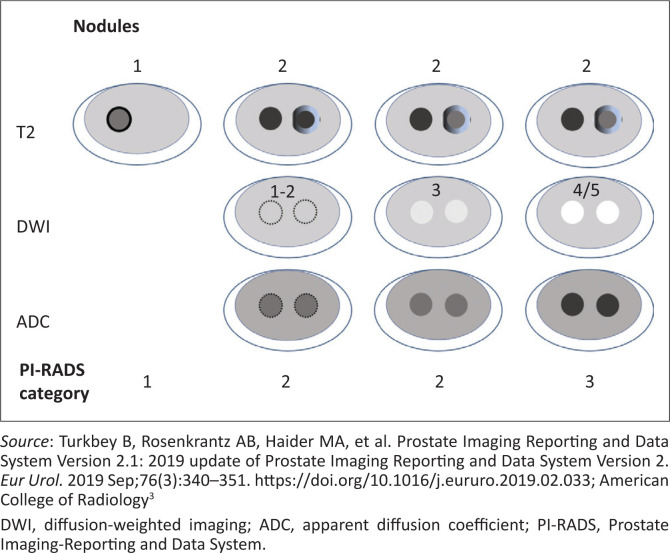
Schematic diagram detailing the multi-sequence evaluation of transition zone nodules including diffusion-weighted imaging. The dotted lines indicate a near-isointense lesion with indistinct borders.^10^

Interpreting the TZ is always challenging in the presence of BPH with the presence of hyperplastic nodules, cystic changes and both glandular and stromal components of mixed T2 signal intensities. T2 imaging remains the *dominant* sequence with DWI-ADC the complimentary sequence. Always assess the TZ in at least two planes as morphological assessment of nodules and the background tissue is crucial (Tables 5, 6 and Figures 5–7).

**TABLE 5:** Characterisation and scoring of a lesion in the TZ.

Score	Findings
1	Normal appearing TZ (rare) or homogeneous, round and completely encapsulated nodule, that is, ‘Typical Nodule’.
2	Mostly encapsulated nodule or homogeneous round, partially encapsulated or non-encapsulated nodule (‘Atypical Nodule’) or a homogeneous mildly hypointense area between nodules.
3	Heterogeneous or poorly defined nodule; that is heterogeneous signal intensity with obscured margins; includes others that are NOT 2, 4 or 5.
4	Lenticular. Non-circumscribed, homogeneous, moderately hypointense ≤ 1.5 cm in greatest dimension.
5	As above. ≥ 1.5 cm in greatest dimension or EPE or invasive behaviour.

*Source*: Turkbey B, Rosenkrantz AB, Haider MA, et al. Prostate Imaging Reporting and Data System Version 2.1: 2019 update of Prostate Imaging Reporting and Data System Version 2. *Eur Urol.* 2019 Sep; 76(3):340–351. https://doi.org/10.1016/j.eururo.2019.02.033; American College of Radiology^3^

Note: T2 is the main sequence and diffusion the complimentary sequence.^3^

TZ, transition zone; EPE, extra-prostatic extension.

**TABLE 6:** Combined scoring system to evaluate a TZ lesion.

T2 score dominant sequence	DWI score secondary sequence	DCE score	Overall PI-RADS v2.1 score
1	Any	Any	1
2	≤ 3	Any	2
2	≥ 4	Any	3
3	≤ 4	Any	4
3	5	Any	4
4	Any	Any	4
5	Any	Any	5

*Source*: Turkbey B, Rosenkrantz AB, Haider MA, et al. Prostate Imaging Reporting and Data System Version 2.1: 2019 update of Prostate Imaging Reporting and Data System Version 2. *Eur Urol.* 2019 Sep; 76(3):340–351. https://doi.org/10.1016/j.eururo.2019.02.033; American College of Radiology^3^

TZ, transition zone; DWI, diffusion-weighted imaging; DCE, dynamic contrast enhanced; PI-RADS, Prostate Imaging-Reporting and Data System.

T2 morphology: shape of lesion especially if lenticular. Marginal features: See Figure 6 and 7.

- Completely encapsulated nodule (score 1).
- No capsule, homogeneous circumscribed nodule (score 2).
- Majority encapsulated circumscribed nodule (score 2).

Prostate Imaging-Reporting and Data System v2.1 acknowledges that the age demographics of men undergoing MRI to exclude/confirm PCa will have an increased incidence of BPH. It therefore *RECLASSIFIES* the classic appearing BPH nodules (encapsulated) from PI-RADS score 2 (previous) to PI-RADS score 1. Score 2 is now reserved for ‘atypical nodules’, that is, homogeneously circumscribed, partially encapsulated nodules or homogeneous mildly hyperintense areas between nodules (previously would have been scored 3). Microcysts within nodules due to dilated hyperplastic acini are benign changes (score 1 or 2).

#### Nodules with scores of 2 or more must be further evaluated with diffusion-weighted imaging and ADC

If a score of 3 is established, then DWI-ADC is used to assign a definitive PI-RADS score. DWI-ADC images should be interpreted using PZ criteria. See combined scoring system in Table 6.

If on T2W images, a lesion has a score of 2, then:

- DWI-ADC score 1–3 assigns established PI-RADS score of 2.
- DWI-ADC score 4–5 assigns established PI-RADS score of 3.

If on T2W images, a lesion has a score of 3, then:

- DWI-ADC score 1–4 assigns a definitive PI-RADS score of 3.
- DWI-ADC score 5 assigns a definitive PI-RADS score of 4.

### Central zone interpretation

**FIGURE 8:**
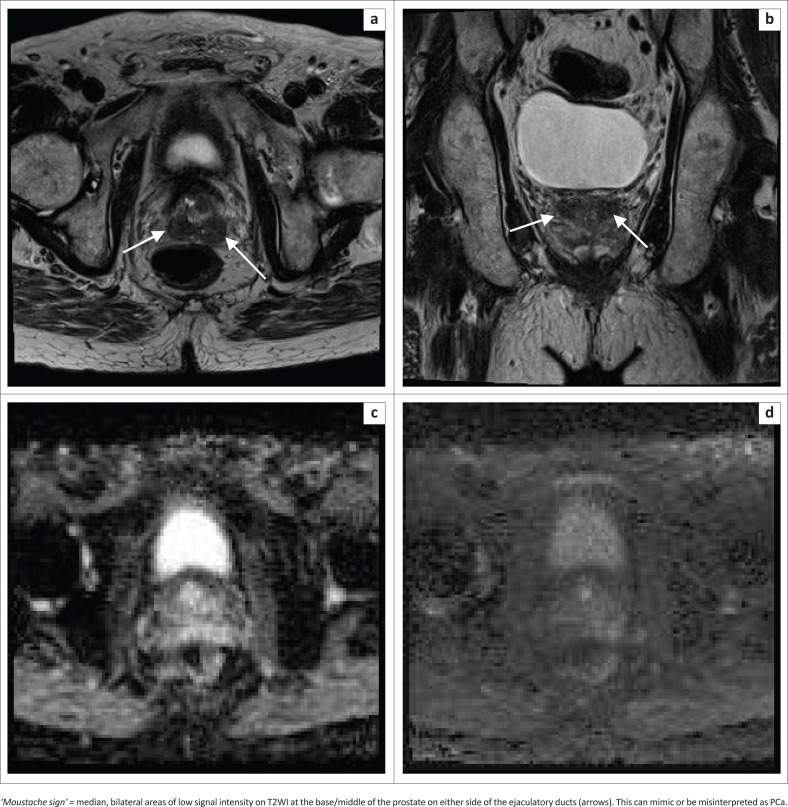
Central zone (a, b, c and d). At the prostate base the central zone is homogeneously T2 hypointense.

A normal CZ is seen in 93% of MRI studies.^1^ It is recognised as low T2 signal intensity tissue surrounding the ejaculatory ducts, posterior to the TZ at the prostatic base, coursing medially to the urethra at the level of the verumontanum. The CZ is normally symmetrical and at high *b*-value DWI, is symmetrically mildly hyperintense. It does not demonstrate early enhancement on DCE images and has Type 1 enhancement.^1^ See Figure 8.

#### Key pointers

Symmetry at base level:

- Can be compressed by the TZ (especially if hyperplastic) posteriorly.
- ‘Teardrop’ or ‘moustache’ shape.^1^

Beware: can appear asymmetric in 18%.^1^

Prostate cancer can occur in the CZ in 5%. It tends to be more aggressive with a higher grade and increased incidence of EPE and seminal vesicle invasion.^1^

Suspect tumour when there is asymmetry, and the lesion extends beyond the verumontanum, especially if there is a mass-like change. Dynamic contrast enhanced imaging is helpful. Central zone tumours demonstrate early enhancement with type 3 (wash-out) curves, compared to the normal CZ which has a type 1 wash-out curve, that is, progressive enhancement.^1,11,12^

### Anterior fibromuscular stroma interpretation

**FIGURE 9:**
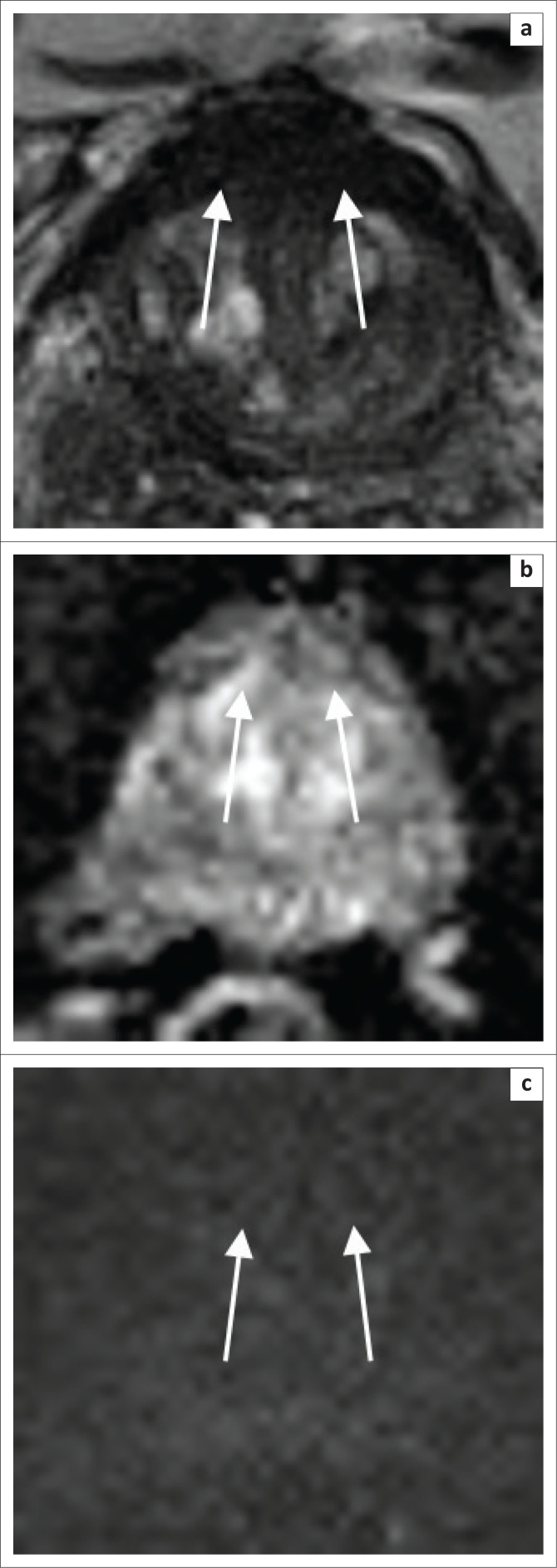
The anterior fibromuscular stroma shows marked homogeneously T2-hypointense signal (a). It is ADC hypointense (b) and diffusion-weighted imaging iso-intense (c).

Normal AFMS has a bilaterally symmetric shape (crescentic) and symmetrically low SI (equivalent to SI of the pelvic floor muscles) on T2W, ADC and high *b*-value DWI, that is, ‘low on all sequences’.^1^ See Figure 9.

Prostate cancer does not arise in the AFMS but extends from either the PZ or the TZ. Abnormalities in the AFMS with increased SI and restricted diffusion when compared with pelvic floor muscles, asymmetric enlargement or a focal mass, increases the suspicion for tumour from the adjacent PZ or TZ. Determine (may not always be possible) from where the tumour most likely arises and apply the scoring criteria for that area.^1^

### Assessment of extra-prostatic extension

- Frank capsular breech by a suspicious lesion.
- Must include possible invasion of rectum and bladder.
- Broad capsular contact (10 mm – 20 mm).
- Bulging capsule.
- Irregular, spiculated, angular prostate margin adjacent to tumour.
- Asymmetry, traction, thickening of neuro-vascular bundles.
- Seminal vesicle invasion – asymmetric loss of normal T2 hyperintensity in the seminal vesicle lumen.

## Pitfalls

An extruded hyperplastic nodule from the TZ into the PZ, can masquerade as a PZ lesion.

A neurovascular bundle that is close to the capsule or projects into the PZ with false restriction of diffusion can be falsely interpreted as a possible PZ lesion. Prostatitis can be falsely interpreted as a PZ lesion. Atypical anatomy of the central zone can masquerade as a lesion.

## Reporting template

**FIGURE 10:**
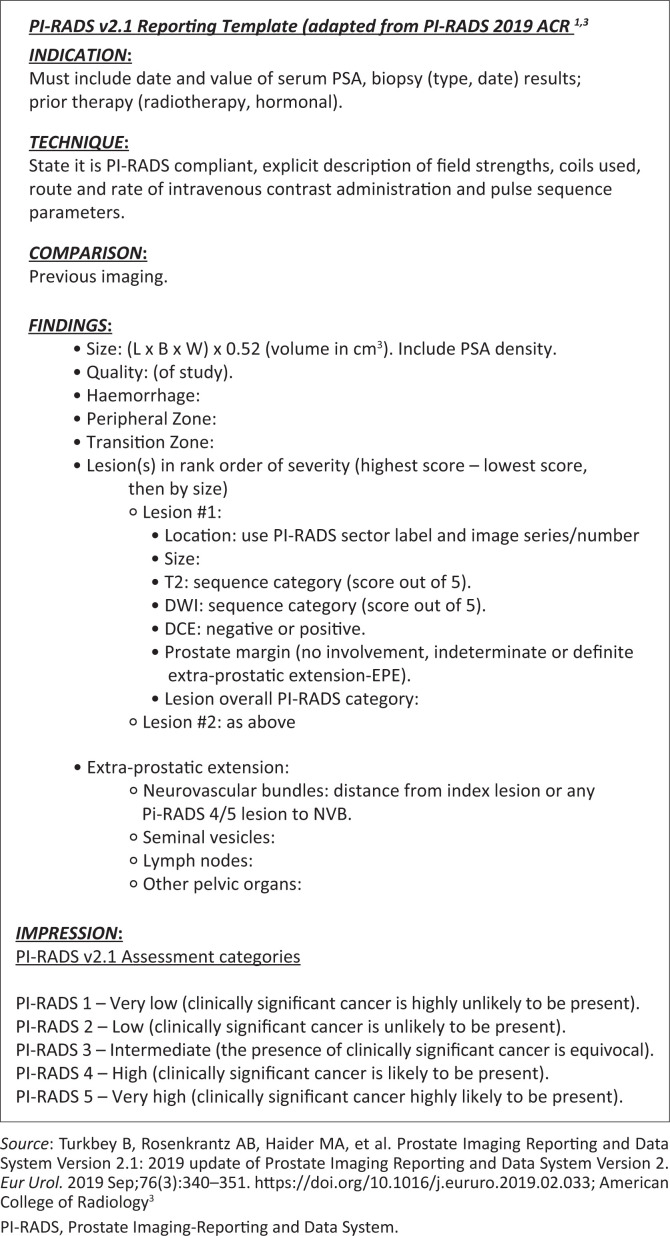
Sample reporting template devised using multiparametric prostate magnetic resonance imaging.

**FIGURE 11:**
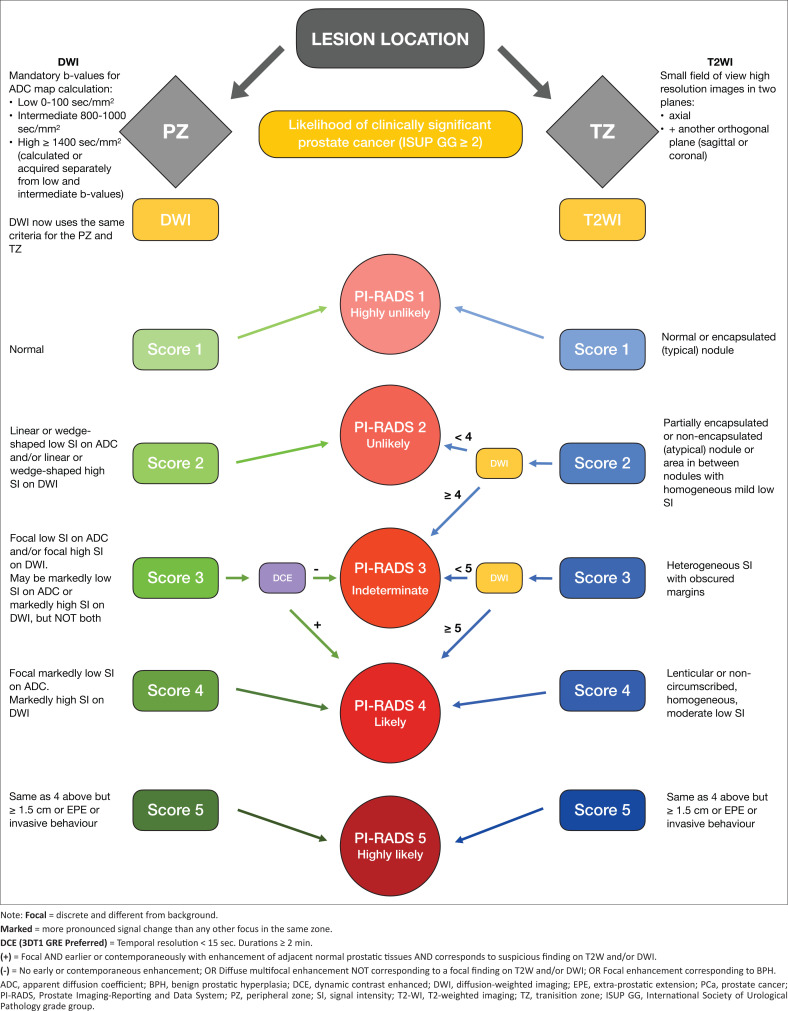
Flow diagram outlining the interplay of findings on relevant multiparametric prostate magnetic resonance imaging sequences for scoring of prostatic lesions in the peripheral zone and transition zone regions.^1,3,10^

See Figure 10 – a template for reporting of multiparametric prostate MRI studies using the algorithm outlined in Figure 11, providing the reporting radiologist with a structured approach to lesions of the peripheral and transitional zones of the prostate.

## Summary

Prostate Imaging-Reporting and Data System v2 is being widely utilised in clinical practice. Worldwide experience has highlighted the areas of ambiguity, poor performance and reduced inter-observer variability, necessitating the upgrade to PI-RADS v2.1, which addresses these issues. There are some minor modifications including a simplified scoring system whilst maintaining the framework for acquisition and interpretation. This fine tuning of a well-established diagnostic imaging system further improves the stratification of risk in patients with suspected prostatic carcinoma. As mpMRI becomes more widely available in developing countries it is expected that the use of risk stratification models, such as PI-RADS will increase. The reporting radiologist who is able to apply the framework of PI-RADS in daily practice will be well-positioned to contribute to the multi-disciplinary management of patients with prostate carcinoma.
